# In vivo staging of frontotemporal lobar degeneration TDP-43 type C pathology

**DOI:** 10.1186/s13195-020-00600-x

**Published:** 2020-03-27

**Authors:** Martina Bocchetta, Maria del Mar Iglesias Espinosa, Tammaryn Lashley, Jason D. Warren, Jonathan D. Rohrer

**Affiliations:** 1 Dementia Research Centre, Department of Neurodegenerative Disease, UCL Queen Square Institute of Neurology, University College London, 8-11 Queen Square, London, WC1N 3BG UK; 2 Department of Neurology, Unidad de Neurología, Hospital Torrecárdenas, Almería, Spain; 3 Queen Square Brain Bank for Neurological Disorders, UCL Queen Square Institute of Neurology, University College London, London, UK; 4 Department of Neurodegenerative Disease, UCL Queen Square Institute of Neurology, University College London, London, UK

**Keywords:** TDP-43 type C, Semantic variant PPA, Magnetic resonance imaging, Disease progression

## Abstract

**Background:**

TDP-43 type C is one of the pathological forms of frontotemporal lobar degeneration (FTLD) and mainly associated clinically with the semantic variant of primary progressive aphasia (svPPA). We aimed to define in vivo the sequential pattern of neuroanatomical involvement in a cohort of patients with FTLD-TDP type C pathology.

**Methods:**

We extracted the volumes of a set of cortical and subcortical regions from MRI scans of 19 patients with *post mortem* confirmed TDP-43 type C pathology (all with left hemisphere-predominant atrophy at baseline). In the initial development phase, we used w-scores computed from 81 cognitively normal controls to define a set of sequential stages of neuroanatomical involvement within the FTLD-TDP type C cohort where a w-score of < − 1.65 was considered abnormal. In a subsequent validation phase, we used 31 follow-up scans from 14 of the 19 patients in the same cohort to confirm the staging model.

**Results:**

Four sequential stages were identified in the initial development phase. *Stage 1* was defined by atrophy in the left amygdala, medial temporal cortex, temporal pole, lateral temporal cortex and right medial temporal cortex; *Stage 2* by atrophy in the left supratemporal cortex; *Stage 3* by atrophy in the right anterior insula; and *Stage 4* by atrophy in the right accumbens. In the validation phase, calculation of w-scores in the longitudinal scans confirmed the staging system, with all patients either staying in the same stage or progressing to a later stage at follow-up.

**Conclusion:**

In vivo imaging is able to detect distinct stages of neuroanatomical involvement in FTLD-TDP type C pathology. Using an imaging-derived staging system allows a more refined stratification of patients with svPPA during life.

## Introduction

Frontotemporal lobar degeneration (FTLD) is a heterogenous disease, both clinically, genetically and pathologically. About half of all FTLD cases have inclusions of the TAR DNA-binding protein 43 (TDP-43), which can be subdivided into five different subtypes: A, B, C, D and E [1, 2]. Little is currently known about the sequential pattern of neuroanatomical involvement in TDP-43 proteinopathies, in part due to the fact that individually these are rare neurodegenerative diseases. Whilst one previous study has described the progressive pattern of distribution of TDP-43 in the brain at *post mortem* in patients with behavioural variant frontotemporal dementia (FTD), the study included patients with multiple different subtypes of TDP-43 [3].

FTLD-TDP type C is associated most commonly with a clinical diagnosis of semantic dementia [4], now more commonly called semantic variant primary progressive aphasia (svPPA), a disorder that presents with anomia and impaired single-word comprehension [5]. All patients show an asymmetric pattern of atrophy, predominantly localised in the temporal lobe [4], but this can be either left or right hemisphere predominant [6, 7]. When atrophy starts in the right hemisphere, the initial diagnosis can be behavioural variant FTD, although semantic deficits often then develop later [8].

Very few longitudinal studies [9–14] have investigated the evolution of brain changes in svPPA, and to our knowledge, no study has systematically explored this in a patient group with confirmed TDP-43 type C pathology alone.

In this study, we aimed to investigate in vivo the sequential pattern of atrophy in a cohort of patients with TDP-43 type C pathology and identify progressive disease stages.

## Methods

We reviewed the UCL Dementia Research Centre FTD MRI database to identify patients with *post mortem* confirmation of TDP-43 type C pathology, and a good quality T1-weighted magnetic resonance (MR) scan. Nineteen patients were identified [mean (standard deviation) age at baseline 64.5 (6.8) years; 68% male] with 14 also having follow-up imaging (5 with two scans, 5 with three, 1 with four, 2 with five and 1 with six). Eighty-one age- and gender-matched cognitively normal participants with a good quality volumetric T1-weighted MRI were identified as controls [mean (standard deviation) age at scan 60.4 (12.6) years; 43% male]. The study was approved by the local ethics committee, and written informed consent was obtained from all participants.

All patients had initially undergone a standard assessment in the Specialist Cognitive Disorders Clinic at the National Hospital for Neurology and Neurosurgery by a cognitive neurologist, and all had received a diagnosis of semantic dementia. Symptom onset was defined by when the patient and/or their informant (usually their partner or family member) reported the development of the first abnormality, which for all patients was word-finding difficulty. Cognitive assessment usually included a Mini-Mental State Examination (MMSE) [15], verbal IQ (VIQ) and performance IQ (PIQ) from the Wechsler Adult Intelligence Scale Third Edition (WAIS-III) and the Graded Naming Test (GNT) [16]. Of note, all 19 patients had left temporal-predominant atrophy at baseline on their MR imaging.

T1-weighted MRIs of patients and controls were acquired from 1992 to 2014 with scanners from three different manufacturers: 87 on 1.5-T Signa MRI scanner (GE Medical systems, Milwaukee, WI, TR = 12 ms, TI = 650 ms, TE = 5 ms, acquisition matrix = 256 × 256, spatial resolution = 1.5 mm) and 45 on 3-T Trio MRI scanner (Siemens, Erlangen, Germany, TR = 2200 ms, TI = 900 ms, TE = 2.9 ms, acquisition matrix = 256 × 256, spatial resolution = 1.1 mm).

**Fig. 1:**
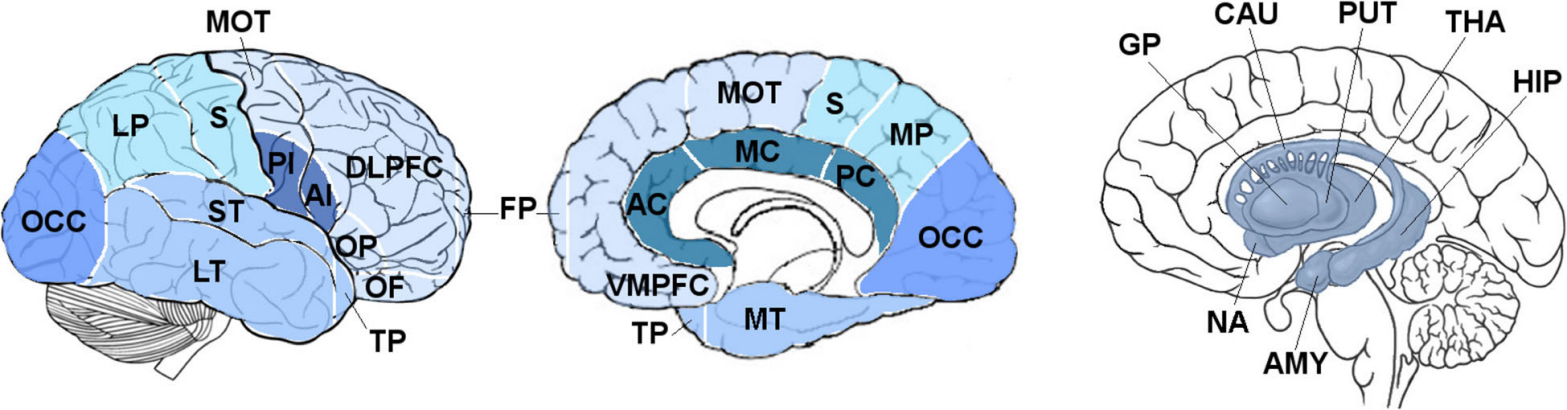
Regions of interest used in the staging analysis. Abbreviations: THA thalamus, PUT putamen, GP pallidum, CAU caudate, NA nucleus accumbens, HIP hippocampus, AMY amygdala, OCC occipital, LP lateral parietal, S sensory, PI posterior insular, AI anterior insular, LT lateral temporal, ST supratemporal, TP temporal pole, DLPFC dorsolateral prefrontal, OP opercular, OF orbitofrontal, FP frontal pole, VMPFC ventromedial prefrontal, AC anterior cingulate, MC middle cingulate, PC posterior cingulate, MT medial temporal, MP medial parietal, MOT motor

Volumetric MRI scans were first bias field corrected and whole-brain parcellated using the geodesic information flow (GIF) algorithm [17], which is based on atlas propagation and label fusion. We extracted volumes of the nineteen cortical regions from GIF (Fig. 1): six frontal (dorsolateral and ventromedial prefrontal, orbitofrontal, motor, opercular, frontal pole), four temporal (medial, lateral, supratemporal, temporal pole), three parietal (medial, lateral, sensory), anterior and posterior insular, cingulate (anterior, middle and posterior) and occipital. We also extracted volumes of subcortical structures (pallidum, putamen, caudate, nucleus accumbens and thalamus) (Fig. 1). To obtain a better segmentation of the hippocampus and amygdala in a cohort of patients with such atrophic medio-temporal regions, the volumes of these two structures were obtained using a customised version of the module available in FreeSurfer 6.0 [18, 19], to adapt the output of GIF to the FreeSurfer format (Fig. 1).

Total intracranial volume (TIV) was computed with SPM12 v6470 (Statistical Parametric Mapping, Wellcome Trust Centre for Neuroimaging, London, UK) running under Matlab R2014b (Math Works, Natick, MA, USA) [20]. All segmentations were visually quality checked.

For each patient’s visit, we computed the w-scores of the brain volumes from controls, using the following formula: *w-score = [(observed volume in patient) – (predicted patient volume)]/(square root of the residual variance)*, where the predicted patient volume and the residual variance in controls were estimated from a linear regression model carried out on the volumes of the controls adjusting for the effect of age, gender, TIV and scanner type. w-scores lower than − 1.65 (corresponding to the 5th percentile of the controls) were considered as indicative of abnormally small volumes.

The staging system was created by identifying a set of brain regions in which patients had a w-score of less than − 1.65, *only* within that region *or* in the region of an earlier stage, i.e. the first stage required all 19 patients to have a w-score of less than − 1.65 in that stage-defining region *but not* the regions defining later stages, whilst in the second stage, patients required a w-score of less than − 1.65 in that stage-defining region *and* the region defining the prior stage, *but not* in the regions defining later stages. This methodology was continued until no further stages could be defined.

In order to assess whether the initially developed staging system was valid in a separate set of MRI scans, we used the 31 follow-up images available from the same set of patients to see whether the identified brain regions continued to identify distinct stages in the same way, i.e. there were no scans that had a w-score of less than − 1.65 in a later stage but not an earlier stage.

Statistical analyses were performed in Stata v.14 (Stata Statistical Software: College Station, TX: StataCorp LP) and SPSS v. 22 software (SPSS Inc., Chicago, IL, USA).

## Results

### Development of the staging system

The w-score was < − 1.65 in all 19 patients in five regions: the left amygdala, left medial temporal cortex, left temporal pole, left lateral temporal cortex and right medial temporal cortex—these regions then defined *Stage 1*. w-scores in the left supratemporal cortex were less than − 1.65 in 17 patients, hence defining *Stage 2*, whilst w-scores in the right anterior insula were less than − 1.65 in 6 patients (*Stage 3*) and the w-score in the right accumbens was less than − 1.65 in 1 patient (*Stage 4*). Overall, that meant that 2 patients fell into *Stage 1*, 11 in *Stage 2*, 5 in *Stage 3* and 1 in *Stage 4* (Table 1).

**Table 1:**
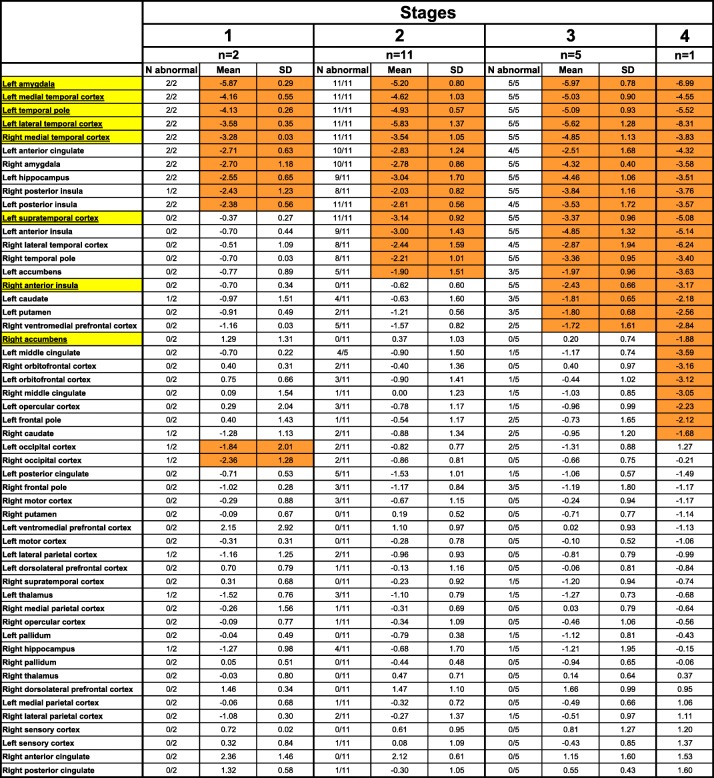
Staging system of FTLD-TDP type C with all cortical and subcortical regions used in the analysis shown by mean w-score at each stage. Yellow denotes the regions chosen to define the stages, and orange denotes abnormal w-scores. Values denote mean and standard deviation (SD) for the w-scores. “n” is the number of cases falling into each stage. “N abnormal” is the number of cases with a w-score < − 1.65 in each stage for each region. Note that the abnormal mean occipital volume in *Stage 1* is driven by a single patient with a lower quality segmentation and therefore likely to be artefactual

**Fig. 2:**
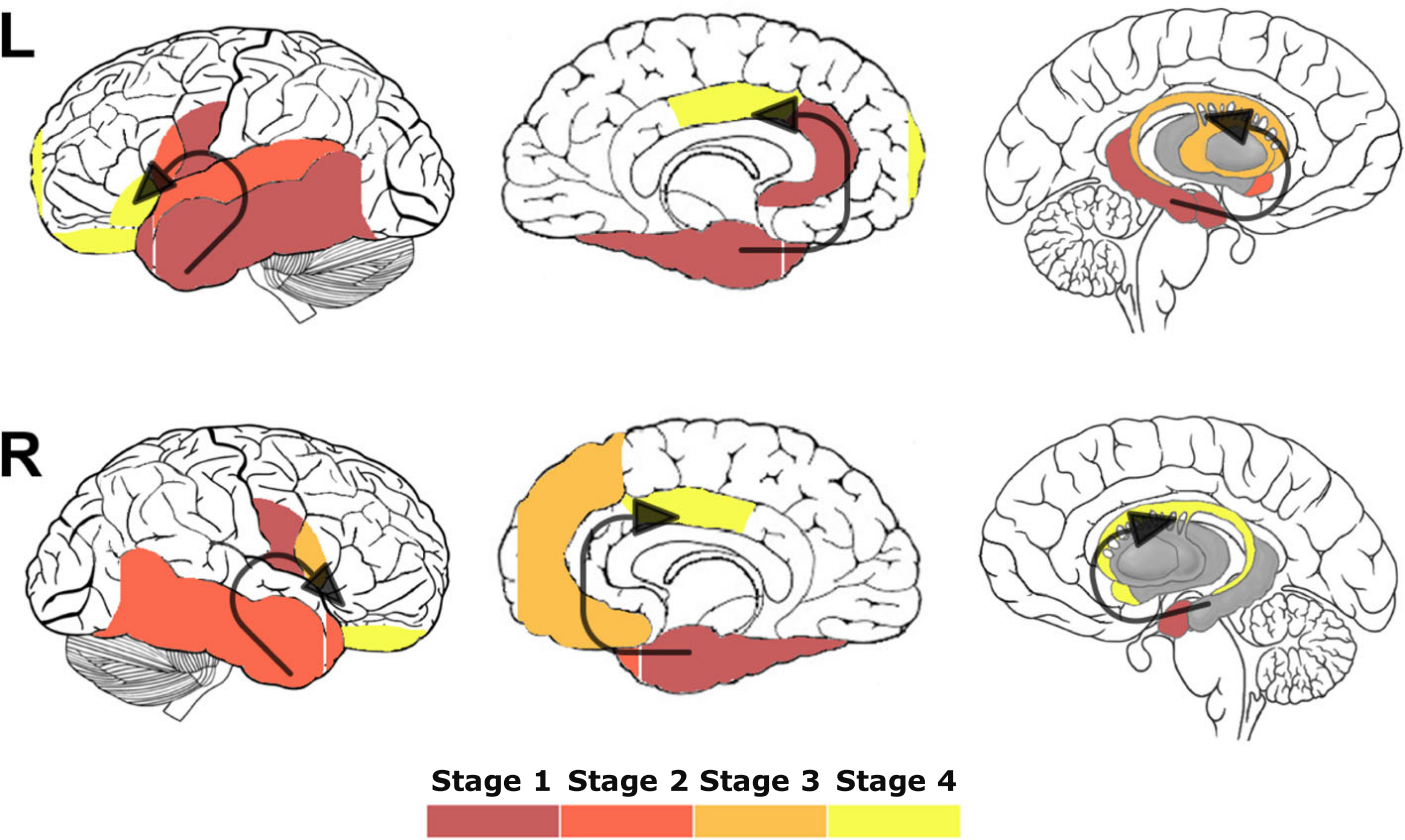
Sequential pattern of neuroanatomical involvement in FTLD-TDP type C. The colour map indicates the stage when the specific region of interest becomes involved

We then reviewed which regions had a mean w-score of less − 1.65 within each stage (even if not every patient within that stage individually had a w-score of less than − 1.65) (Table 1 and Fig. 2).

#### *Stage 1*

 As well as the stage-defining regions of the left amygdala [mean (standard deviation), − 5.9 (0.3)], left medial [− 4.2 (0.6)] and lateral temporal cortex [− 3.6 (0.4)], the left temporal pole [− 4.1 (0.3)] and the right medial temporal cortex [− 3.3 (0.03)], other regions with a mean w-score of < − 1.65 were the left anterior cingulate [− 2.7 (0.63)], hippocampus [− 2.6 (0.65)] and posterior insula [− 2.4 (0.56)] and the right amygdala [− 2.7 (1.18)] and posterior insula [− 2.4 (1.23)]. A mean w-score of < − 1.65 was also found in the right and left occipital cortices [− 2.4 (1.28) and − 1.8 (2.01)], although this was driven by a single patient with a lower quality segmentation of the occipital cortex.

#### *Stage 2*

 As well as the stage-defining region of the left supratemporal cortex [− 3.1 (0.9)], two other regions on the left had a mean w-score of − 1.65: the anterior insula [− 3.0 (1.4)] and the accumbens [− 1.9 (1.5)], as well as two regions on the right, the lateral temporal cortex [− 2.4 (1.6)] and the temporal pole [− 2.2 (1.0)].

#### *Stage 3*

 As well as the stage-defining region of the right anterior insula [− 2.4 (0.7)], regions with a mean w-score of − 1.65 were the left caudate [− 1.8 (0.7)] and putamen [− 1.8 (0.7)] and the right ventromedial prefrontal cortex [− 1.7 (1.6)].

#### *Stage 4*

 As well as the stage-defining region of the right accumbens [− 1.9], which was spared in the other patients, abnormal mean w-scores were found in the left middle cingulate [− 3.6], frontal pole [− 2.1], opercular [− 2.2] and orbitofrontal cortex [− 3.1], and the right orbitofrontal cortex [− 3.2], middle cingulate [− 3.1] and caudate [− 1.7].

### Validation of the staging system

Based on the w-scores of the stage-defining regions, we classified the follow-up scans of 14 patients according to the stages they fell in to (Table 2). Importantly, in none of the 31 MRI scans did the initial staging system not work, i.e. there were no scans in which a w-score of − 1.65 in a later stage but not an earlier stage (Table 2).

**Table 2:**
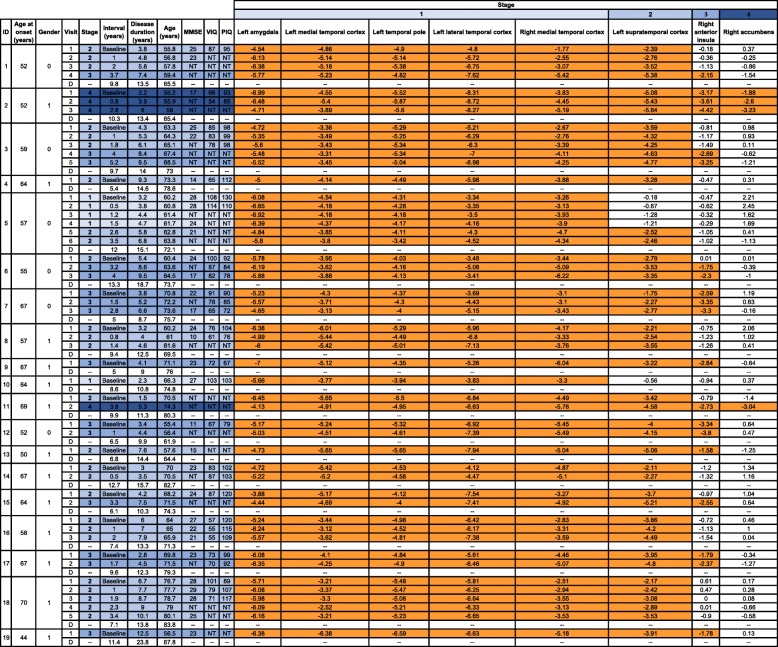
Validation of the stages on follow-up scans of the FTLD-TDP type C cohort. Orange denotes abnormal w-scores. Note that patients with IDs 4, 9, 10, 13 and 19 only had a single scan. MMSE Mini-Mental State Examination, VIQ verbal IQ, PIQ performance IQ, NT not tested. *Interval* from baseline scan to each subsequent visit and to death (D) is shown, as is total *disease duration* at each visit and at time of death (D)

One patient stayed in *Stage 1* (for 1.5 years) and then progressed to *Stage 2* for the next follow-up (at 2.6 years from baseline scan). Four patients remained in *Stage 2* during their follow-up visits (up to 3.4 years from baseline scan), whilst four patients progressed from *Stage 2* to *Stage 3* at follow-up (at 3.2 to 4 years from baseline scan), and another patient progressed from *Stage 2* to *Stage 4* (at 3.8 years from baseline scan). Three patients were in *Stage 3* at baseline and remained in that stage (up to 2.8 years from baseline scan), whilst another patient who was at *Stage 4* at baseline continued in that last stage over the next two follow-up scans (2.8 years from initial scan).

### Disease duration and cognitive symptoms at each stage

Mean (standard deviation) disease duration (based on patient or informant-reported symptom onset) was 3.7 (1.0) in *Stage 1* (range 2.3–4.7 years), 5.9 (2.2) in *Stage 2* (range 1.5–10.1 years), 6.5 (2.8) in *Stage 3* (range 2.8–12.5 years) and 4.6 (1.3) in *Stage 4* (range 3.2–6.0 years).

Cognitively, even those at *Stage 1* were profoundly anomic, with all patients scoring below the 5th percentile on the GNT. Mean (standard deviation) MMSE at *Stage 1* was 26.8 (1.9), at *Stage 2* was 22.6 (4.9), at *Stage 3* was 20.4 (5.3) and at *Stage 4* was 17.0. Mean (standard deviation) VIQ and PIQ were respectively 108.3 (5.5) and 114.3 (14.0) at *Stage 1*, 77.1 (14.5) and 103.9 (11.3) at *Stage 2*, 76.1 (9.0) and 82.9 (10.1) at *Stage 3* and 60.0 (8.5) and 89.0 (5.7) at *Stage 4*.

We also performed correlation analyses between the cognitive scores and the volumes of the stage-defining regions. VIQ significantly correlated with the left lateral temporal cortex (Spearman’s rho = 0.709, *p* value < 0.0005), left supratemporal cortex (0.660, < 0.0005), right anterior insula (0.421, 0.018) and right accumbens (0.394, 0.028) volumes, i.e. regions in all four stages. PIQ was significantly correlated with the right anterior insula (0.607, < 0.0005) and right accumbens (0.466, 0.008) volumes, i.e. only regions affected in *Stages 3* and *4*. MMSE was significantly correlated with the right medial temporal cortex (0.517, 0.003), left supratemporal cortex (0.493, 0.005) and right anterior insula (0.721, < 0.0005) volumes, i.e. only regions affected in *Stage 1* to *3*, but not *4*.

## Discussion

Using w-scores of regional brain volumes, we have characterised four stages of TDP-43 type C disease, describing the progressive pattern of brain involvement in this specific pathological entity and confirming the validity of this staging system by applying it to longitudinal imaging within the cohort.

There are currently no staging systems that address the specific subtypes of TDP-43 pathology. Pathological staging of rare neurodegenerative diseases is difficult as most people who come to *post mortem* are at a very late stage—few people die of another cause at an early stage in the illness. Nonetheless, a staging of TDP-43 pathology in behavioural variant FTD has been suggested, with spread from an earlier stage in the orbitofrontal lobes and basolateral amygdala through the frontal and temporal lobes to later stages in the parietal and occipital lobes [3]. However, firstly, this study combines TDP-43 types A, B and C (including those with *C9orf72* and *GRN* mutations) together within the same staging system, and secondly, it describes a completely symmetrical pattern of disease spread. Evidence from in vivo imaging studies which show distinct patterns of neuroanatomical involvement in each of the TDP-43 subtypes, with both types A and C usually showing markedly asymmetric atrophy [6, 7], suggests that a combined staging system is unlikely to describe the complete picture of what is happening with disease progression. Our study aims to address this by focusing on one form of TDP-43 pathology.

One of the major issues with any staging system is capturing patients early enough in the disease process. At the earliest stage in our study, all patients showed atrophy in the left medio-lateral temporal cortex, temporal pole and amygdala and in the right medial temporal cortex. Whilst this is in line with previous reports of patients with early svPPA [8, 11, 21], few studies have ever managed to capture people with minimal atrophy at presentation. In general, most patients with svPPA come to the attention of neurologists a number of years into the illness when atrophy has already become readily apparent. One study managed to capture a very early stage of svPPA when only mild left anteromedial temporal lobe atrophy was present when visually assessed [22], but nonetheless, it will be difficult to dissect our *Stage 1* further into a very early and more established stage, without a better method of capturing more patients earlier in their disease process. This is made even more difficult because of the sporadic nature of the disease, with no clearly identifiable presymptomatic stage.

The study shows that with disease progression, neuroanatomical involvement spreads from the left temporal and anteromedial right temporal lobe to more posterior parts of the left temporal lobe (left supratemporal cortex) and more superior regions (left insula), and in the right temporal lobe to more lateral regions. Following this, subcortical involvement of the striatum is seen in the left hemisphere with more superior spread in the right hemisphere (left insula). In the last stage, there is now subcortical involvement in the right hemisphere, and more anterosuperior and basal frontal regions in both hemispheres. This is consistent with previous longitudinal studies of svPPA [8–14, 21, 23].

Only left temporal-predominant cases were included in the study, as these were the only cases in our database. Right temporal-predominant disease is reported to a lesser extent than the left temporal variant [24], but this is likely to represent a reporting bias, potentially as patients with a more behavioural or ‘psychiatric’ symptom onset present less frequently to neurologists. However, prior studies have suggested that the pattern of brain atrophy in the right temporal variant is the mirror image of that in the left temporal variant [8], suggesting that the staging system developed here may be valid but with stage-defining regions reversed to the opposite hemisphere. Future studies in pathologically confirmed TDP-43 type C right temporal variant disease will be required to confirm this.

Limitations of this study include the relatively small sample size, although for such a rare pathological presentation, it is unlikely that many centres have studied a larger dataset. Secondly, as the patients were seen over a long period of time, a consistent cognitive battery of tests was not performed in every patient. However, all patients had the GNT at baseline, with all scoring in the abnormal range (below the 5th percentile). This is a difficult naming task, but nonetheless, shows that anomia is a very early feature of this disease. Other focal cognitive and behavioural symptom scores were not available for review in this cohort, but disease progression in svPPA is known to be associated with the development of impaired cognition and behaviour, e.g. at an early stage impaired emotional processing and change in appetite are seen [8, 12, 25], features consistent with atrophy in the insular and temporal cortices (*Stage 2*), whilst disinhibition and obsessive-compulsive behaviour typically occur later [8] and are associated with ventromedial and orbitofrontal cortex dysfunction (*Stages 3* and *4* in our model). Future studies will need to incorporate a more detailed review of cognitive and behavioural symptoms seen at each stage [26] and recognise that these will be different for the left and right temporal variants. Lastly, whilst the calculation of w-scores takes into account variability due to age, gender, TIV and scanner type, there could potentially remain some variability between regions of interest due to their different sizes, which would differentially affect their ability to detect a change in volume.

The study highlights the unreliability of patient- and informant-reported symptom onset to define disease duration—wide ranges of disease duration are reported for each stage, and whilst the mean disease duration increases for *Stages 1* to *3* (3.7, 5.9, 6.5), the mean for *Stage 4* was 4.6 years. Similarly, although there is a decrease in mean MMSE of 2 to 4 points per stage, and a significant correlation with regional w-scores implicated in the first three stages, the individual MMSEs within each stage are variable. Hopefully, the current staging system will allow clinicians to better define the stage at which their patient with svPPA is currently in.

## Conclusions

In summary, using MR imaging from a group of patients with *post mortem* confirmed TDP-43 type C pathology, we have defined a staging system which describes the sequential involvement of brain regions as the disease progresses. Using an imaging-derived staging system allows a more refined stratification of patients with svPPA during life. This will allow future studies to test and replicate this system further in different, independent and larger cohorts to understand this specific pathological form of FTLD better. Future studies using different methods, such as voxel-based approaches, will also be important to further test this staging system.

## Data Availability

Anonymised data are available from the corresponding author upon reasonable request.

## References

[1] Mackenzie IR, Neumann M, Baborie A (2011). A harmonized classification system for FTLD-TDP pathology. Acta Neuropathol.

[2] Lee EB, Porta S, Michael Baer G (2017). Expansion of the classification of FTLD-TDP: distinct pathology associated with rapidly progressive frontotemporal degeneration. Acta Neuropathol.

[3] Brettschneider J, Del Tredici K, Irwin DJ (2014). Sequential distribution of pTDP-43 pathology in behavioral variant frontotemporal dementia (bvFTD). Acta Neuropathol.

[4] Rohrer JD, Lashley T, Schott JM (2011). Clinical and neuroanatomical signatures of tissue pathology in frontotemporal lobar degeneration. Brain..

[5] Gorno-Tempini ML, Hillis AE, Weintraub S (2011). Classification of primary progressive aphasia and its variants. Neurology..

[6] Rohrer D, Geser F, Zhou J (2010). TDP-43 subtypes are associated with distinct atrophy patterns in frontotemporal dementia. Neurology..

[7] Whitwell JL, Jack CR, Parisi JE (2010). Does TDP-43 type confer a distinct pattern of atrophy in frontotemporal lobar degeneration?. Neurology..

[8] Seeley WW, Bauer AM, Miller BL (2005). The natural history of temporal variant frontotemporal dementia. Neurology.

[9] Whitwell JL, Anderson VM, Scahill RI, Rossor MN, Fox NC (2004). Longitudinal patterns of regional change on volumetric MRI in frontotemporal lobar degeneration. Dement Geriatr Cogn Disord.

[10] Rohrer JD, McNaught E, Foster J (2008). Tracking progression in frontotemporal lobar degeneration: serial MRI in semantic dementia. Neurology..

[11] Rohrer JD, Warren JD, Modat M (2009). Patterns of cortical thinning in the language variants of frontotemporal lobar degeneration. Neurology..

[12] Brambati SM, Rankin KP, Narvid J (2009). Atrophy progression in semantic dementia with asymmetric temporal involvement: a tensor-based morphometry study. Neurobiol Aging.

[13] Rogalski E, Cobia D, Harrison TM, Wieneke C, Weintraub S, Mesulam MM (2011). Progression of language decline and cortical atrophy in subtypes of primary progressive aphasia. Neurology..

[14] Kumfor F, Landin-Romero R, Devenney E (2016). On the right side? A longitudinal study of left- versus right-lateralized semantic dementia. Brain..

[15] Folstein MF, Folstein SE, McHugh PR (1975). “Mini-mental state”. A practical method for grading the cognitive state of patients for the clinician. J Psychiatr Res.

[16] McKenna P, Warrington EK (1980). Testing for nominal dysphasia. J Neurol Neurosurg Psychiatry.

[17] Cardoso MJ, Modat M, Wolz R, et al. Geodesic information flows: spatially-variant graphs and their application to segmentation and fusion. IEEE TMI. 2015. 10.1109/TMI.2015.2418298. 10.1109/TMI.2015.2418298 25879909

[18] Iglesias JE, Augustinack JC, Nguyen K (2015). A computational atlas of the hippocampal formation using ex vivo, ultra-high resolution MRI: application to adaptive segmentation of in vivo MRI. Neuroimage..

[19] Saygin ZM, Kliemann D, Iglesias JE (2017). High-resolution magnetic resonance imaging reveals nuclei of the human amygdala: manual segmentation to automatic atlas. Neuroimage..

[20] Malone IB, Leung KK, Clegg S (2015). Accurate automatic estimation of total intracranial volume: a nuisance variable with less nuisance. Neuroimage..

[21] Bright P, Moss HE, Stamatakis EA, Tyler LK (2008). Longitudinal studies of semantic dementia: the relationship between structural and functional changes over time. Neuropsychologia.

[22] Czarnecki K, Duffy JR, Nehl CR (2008). Very early semantic dementia with progressive temporal lobe atrophy: an 8-year longitudinal study. Arch Neurol.

[23] Landin-Romero R, Tan R, Hodges JR, Kumfor F (2016). An update on semantic dementia: genetics, imaging, and pathology. Alzheimers Res Ther.

[24] Thompson SA, Patterson K, Hodges JR (2003). Left/right asymmetry of atrophy in semantic dementia: behavioral-cognitive implications. Neurology..

[25] Rosen HJ, Allison SC, Ogar JM (2006). Behavioral features in semantic dementia vs other forms of progressive aphasias. Neurology..

[26] Bocchetta M, Iglesias JE, Russell LL (2019). Segmentation of medial temporal subregions reveals early right-sided involvement in semantic variant PPA. Alzheimers Res Ther.

